# Fucoidan Improves Tumour Control and Liver Function in TACE for Unresectable Hepatocellular Carcinoma: A Randomised Trial

**DOI:** 10.1111/liv.70347

**Published:** 2025-09-17

**Authors:** Yanting Zou, Szu‐Yuan Wu, Wanqin Zhang, Wei Zhang, Xizhong Shen, Xudong Qu, Qunyan Yao

**Affiliations:** ^1^ Department of Gastroenterology and Hepatology, Zhongshan Hospital Fudan University Shanghai China; ^2^ Department of Gastroenterology and Hepatology, Zhongshan Hospital (Xiamen) Fudan University Xiamen China; ^3^ Shanghai Institute of Liver Diseases Shanghai China; ^4^ Graduate Institute of Business Administration, College of Management Fu Jen Catholic University Taipei Taiwan; ^5^ Artificial Intelligence Development Center Fu Jen Catholic University Taipei Taiwan; ^6^ Center for Regional Anesthesia and Pain Medicine, Wan Fang Hospital Taipei Medical University Taipei Taiwan; ^7^ Department of Food Nutrition and Health Biotechnology, College of Medical and Health Science Asia University Taichung Taiwan; ^8^ Big Data Center, Lo‐Hsu Medical Foundation Lotung Poh‐Ai Hospital Yilan Taiwan; ^9^ Division of Radiation Oncology, Lo‐Hsu Medical Foundation Lotung Poh‐Ai Hospital Yilan Taiwan; ^10^ Department of Healthcare Administration, College of Medical and Health Science Asia University Taichung Taiwan; ^11^ Department of Interventional Therapy, Zhongshan Hospital Fudan University Shanghai China

**Keywords:** disease control rate, hepatocellular carcinoma, hepatoprotection, low‐molecular‐weight fucoidan, transarterial chemoembolization

## Abstract

**Background:**

Hepatocellular carcinoma (HCC) is a leading cause of cancer‐related mortality. Transarterial chemoembolization (TACE) is the standard locoregional therapy for unresectable HCC but is limited by high recurrence and hepatic toxicity. Low‐molecular‐weight fucoidan (LMF), a sulfated polysaccharide from brown seaweed, has anticancer and hepatoprotective properties. This study assessed whether LMF enhances tumour response and preserves liver function when combined with TACE.

**Methods:**

In this randomised, double‐blind, placebo‐controlled trial, 82 patients with unresectable HCC were randomly assigned (1:1) to receive LMF (4.4 g twice daily) or placebo for 6 months in addition to TACE. Tumour response was assessed using modified RECIST criteria, and liver function was monitored via Child–Pugh classification. The primary endpoint was disease control rate (DCR), with objective response rate (ORR), liver function and adverse events as secondary endpoints.

**Results:**

Baseline characteristics were well balanced. DCR was significantly higher in the LMF group (95.24% vs. 80.00%, *p* = 0.035), with a lower progressive disease rate (4.76% vs. 20.00%). ORR was higher in the LMF group (52.38% vs. 35.00%) but not statistically significant (*p* = 0.1129). LMF preserved liver function (*p* = 0.029), with more patients maintaining Child–Pugh Class A (80.95% vs. 62.50%). Adverse event rates were similar, and no severe adverse events occurred.

**Conclusions:**

LMF improved tumour control, preserved liver function and had a favourable safety profile. These findings suggest LMF may mitigate TACE‐related hepatic toxicity and prolong treatment eligibility, warranting further validation in larger trials.

**Abbreviations**
AEs: adverse events
AFP: alpha‐fetoprotein
ALT: alanine aminotransferase
AST: aspartate aminotransferase
BCLC: Barcelona Clinic Liver Cancer
CI: confidence interval
CR: complete response
CT: computed tomography
CTCAE: Common Terminology Criteria for Adverse Events
DCR: disease control rate
ECOG: Eastern Cooperative Oncology Group
GGT: gamma–glutamyl transferase
HBV: hepatitis B virus
HCC: hepatocellular carcinoma
HCV: hepatitis C virus
HR: hazard ratio
INR: international normalised ratio
LMF: low‐molecular‐weight fucoidan
mRECIST: modified Response Evaluation Criteria in Solid Tumours
NAFLD: nonalcoholic fatty liver disease
ORR: objective response rate
OS: overall survival
PD: progressive disease
PR: partial response
QoL: quality of life
RCT: randomised controlled trial
SAEs: serious adverse events
SD: stable disease
SIRT1: sirtuin 1
TACE: transarterial chemoembolization
TAE: transarterial embolisation
TBil: total bilirubin
TGF‐β1: transforming growth factor beta 1
TKI: tyrosine kinase inhibitor
ULN: upper limit of normal
VEGF: vascular endothelial growth factor
WBC: white blood cell

**Summary**

- DCR was significantly higher in the LMF group (95.24% vs. 80.00%, *p* = 0.035), suggesting enhanced tumour control.
- LMF preserved liver function, as indicated by a significantly higher proportion of patients maintaining Child–Pugh class A status compared with placebo (*p* = 0.029).
- No severe adverse events (SAEs) were reported, and the incidence of adverse events was comparable between groups.

## 1 Introduction

Hepatocellular carcinoma (HCC) is the most common primary liver malignancy, accounting for approximately 80% to 90% of liver cancer cases worldwide [1]. It is the sixth most frequently diagnosed cancer and the third leading cause of cancer‐related mortality, with an estimated 900 000 new cases and more than 830 000 deaths reported in 2020 [2]. The global burden of HCC continues to rise, particularly in regions such as North America, Latin America and Europe [2]. However, the disease remains most prevalent in Asia, where approximately 72% of cases occur, followed by Europe (10%), Africa (8%) and Latin America (5%) [2]. The incidence is highest in Mongolia, with a reported rate of 93.7 cases per 100 000 individuals, while North America has an incidence of 18.3 per 100 000 [3]. HCC exhibits a strong male predominance, with a male‐to‐female ratio of approximately 3:1 [2]. Racial and ethnic disparities have also been noted, particularly in the United States, where the incidence is significantly higher among Asian/Pacific Islanders compared with other population groups [4].

For patients with unresectable HCC, treatment options are guided by disease burden, liver function and patient performance status [5, 6, 7, 8, 9]. Liver transplantation remains the only curative treatment for a proportion of HCC cases, but its application is limited by organ availability and strict eligibility criteria [5, 6]. Locoregional therapies, such as radiofrequency ablation (RFA) and microwave ablation, are effective for small tumours but are not suitable for patients with larger lesions or multifocal disease [5, 6, 10, 11]. For patients who are ineligible for both resection and local ablation, transarterial therapies remain the primary treatment strategy. Transarterial chemoembolization (TACE) is the standard of care for patients with intermediate‐stage HCC who are not candidates for curative therapies [5, 6, 10, 11]. TACE involves the intra‐arterial infusion of chemotherapy, typically emulsified in lipiodol, followed by arterial embolization to enhance drug retention and induce tumour ischemia [12, 13]. A related approach, transarterial embolization (TAE), also known as bland embolization, omits the chemotherapeutic agent and relies solely on embolic agents to obstruct the tumour's blood supply [12, 13]. While TAE has demonstrated efficacy in selected cases, TACE remains the preferred approach due to its superior tumour control and improved survival outcomes [14]. TACE is a widely utilised locoregional therapy for patients with unresectable HCC [5, 6, 7, 8]. While TACE has been shown to improve survival compared to best supportive care, its efficacy as a monotherapy remains suboptimal, with high rates of tumour recurrence and progression [15]. Consequently, there is a critical need to explore adjunctive treatments that can enhance the therapeutic outcomes of TACE [15].

Low‐molecular‐weight fucoidan (LMF), a sulfated polysaccharide derived from brown seaweed, has demonstrated multiple anticancer properties, including inhibition of tumour growth and metastasis, modulation of immune responses, suppression of tumour angiogenesis and enhancement of chemotherapy efficacy [1, 16, 17, 18, 19]. Preclinical and clinical studies suggest that LMF can potentiate the cytotoxic effects of chemotherapy and inhibit hepatocarcinogenesis through pathways such as ASGR/STAT3/HNF4A signalling [19, 20, 21]. However, its potential role as an adjunct to TACE in the treatment of HCC has not been thoroughly investigated. Given its promising anticancer properties, we conducted this randomised controlled trial (RCT) to evaluate whether the addition of LMF to TACE could improve clinical outcomes in patients with unresectable HCC. In the present study, all patients had disease that was unsuitable for surgical resection or local ablative therapy, making TACE the only viable locoregional treatment option. Given the established role of TACE in this setting, the addition of LMF was investigated as a potential strategy to enhance tumour response and improve clinical outcomes. While transarterial therapies remain the cornerstone of treatment for unresectable HCC, ongoing research is needed to refine patient selection, optimise combination strategies and explore novel therapeutic adjuncts that may improve efficacy and long‐term survival.

## 2 Data and Methods

### 2.1 Study Population

This prospective, randomised, double‐blind, placebo‐controlled trial enrolled patients from Zhongshan Hospital, affiliated with Fudan University. The trial was registered under ClinicalTrials.gov ID NCT04066660. Eligible participants were between 18 and 80 years of age with histologically or clinically confirmed unresectable HCC and measurable disease as defined by the modified RECIST criteria. Patients were required to have an Eastern Cooperative Oncology Group (ECOG) performance status of 0 to 2, to have completed any prior local therapy at least 6 weeks before enrollment and to exhibit no acute toxicity of grade 1 or higher per the Common Terminology Criteria for Adverse Events (CTCAE). To ensure homogeneity in treatment response, only patients who had not previously received TACE were included in this study.

To be eligible, patients were required to have Child–Pugh Class A liver function and meet specific laboratory thresholds, including a white blood cell count of at least 3.0 × 10^9^/L, a neutrophil count of at least 1.5 × 10^9^/L, haemoglobin of at least 85 g/L, a platelet count of at least 100 × 10^9^/L, albumin of at least 28 g/L and a total bilirubin level of no more than 51.3 μmol/L. Additionally, the international normalised ratio (INR) had to be 2.3 or lower, prothrombin time could not exceed 6 s above the control level, creatinine was limited to 1.5 times the upper limit of normal (ULN), and both amylase and lipase levels had to be below 1.5 times ULN.

Patients were excluded if they had metastatic disease or had previously received systemic anticancer therapy for HCC, including tyrosine kinase inhibitors, prior to disease control rate (DCR) assessment, to minimise potential interference with the evaluation. Additionally, patients with severe or uncontrolled medical conditions, including uncontrolled hypertension, active or uncontrolled infections, coronary heart disease, recent gastrointestinal bleeding within 30 days, severe renal impairment requiring dialysis, a history of organ transplantation or HIV infection, were excluded. Patients who had undergone major surgical procedures, open biopsy, or significant traumatic injury within 4 weeks—or minor surgical procedures within 2 weeks—prior to enrolment were also excluded. Baseline demographic and clinical characteristics were systematically recorded for all enrolled participants.

### 2.2 Research Program: Interventions and Assessments

Patients were randomly assigned to the study or control group in a 1:1 ratio, and all TACE procedures were performed by experienced interventional radiologists following a standardised protocol approved by the trial committee to ensure reproducibility across both groups. After superselective catheterisation of tumour‐feeding arteries, cone‐beam CT (CBCT) was routinely performed to confirm catheter position, delineate tumour vascular supply and identify potential parasitic feeders. A fixed chemotherapy regimen was used—epirubicin (20–30 mg) plus oxaliplatin (20–50 mg), with dose adjustments based on patient body weight and clinical status. The agents were thoroughly emulsified with ultrafluid lipiodol, with lipiodol volume determined by the sum of target tumour diameters and capped at 20 mL. Under fluoroscopic monitoring, the lipiodol–chemotherapy emulsion was slowly infused into the tumour vasculature, followed by embolisation with 350–560 μm gelatin sponge particles. Embolisation was continued until a predefined standardised endpoint was reached: complete or near‐complete disappearance of tumour vessels on dynamic fluoroscopy, marked slowing of arterial flow and contrast retention within the tumour bed, while avoiding permanent occlusion of the main arterial trunk or reflux into nontarget vessels.

TACE sessions were repeated on demand, with a minimum 4‐week interval between procedures. During the 6‐month study period, each patient could receive up to three TACE sessions. Decisions regarding additional TACE were based on a composite assessment at 4 to 6 weeks posttreatment, including radiologic tumour response per mRECIST, liver function (Child–Pugh class), tumour marker trends and performance status.

In the study group, patients received 4.4 g of low‐molecular‐weight fucoidan (LMF) powder, derived from Laminaria japonica and manufactured by Hi‐Q Marine Biotech International (Taipei, Taiwan), administered twice daily for 6 months. At the end of treatment, contrast‐enhanced CT/MRI was performed for tumour evaluation, with MRI preferred where feasible. In the control group, patients received 4.4 g of cellulose powder (placebo) twice daily for the same duration.

To ensure treatment adherence, research nurses conducted daily telephone follow‐ups with each participant to verify compliance with the assigned intervention. Throughout the trial, patients underwent scheduled blood examinations, abdominal CT scans/MRI, and assessments of adverse events (AEs), in addition to completing a quality‐of‐life (QoL) questionnaire using the European Organization for Research and Treatment of Cancer QLQ‐C30. Adverse events were graded according to the Common Terminology Criteria for Adverse Events (CTCAE), Version 4.02.

All serious adverse events (SAEs) were reviewed and adjudicated by an independent safety monitoring committee to ensure objective assessment and reporting.

### 2.3 Observation Indicators

The primary endpoint of this study was the disease control rate (DCR), defined as the proportion of patients achieving complete response (CR), partial response (PR) or stable disease (SD). DCR was prospectively selected at the trial design stage based on the clinical treatment objectives for intermediate‐stage (BCLC‐B) HCC [22], where delaying disease progression and preserving hepatic reserve are key goals [23, 24, 25, 26, 27, 28]. DCR incorporates both tumour regression and disease stabilisation, thus aligning with real‐world therapeutic priorities in the TACE setting. From a methodological perspective, DCR allows the detection of clinically meaningful treatment effects within the study's planned follow‐up period and sample size, and its prognostic linkage to PFS and OS is supported by regulatory guidance Mushti et al. [29] and prior HCC literature [23, 24, 25, 26, 27, 28]. Secondary endpoints included the objective response rate (ORR), incidence of adverse events and changes in quality‐of‐life scores. Tumour response assessments were conducted through central radiologic review to ensure consistency and minimise potential investigator bias. Tumour response was assessed using the modified Response Evaluation Criteria in Solid Tumors (mRECIST), which was applied to evaluate target lesions before and after treatment, thereby determining both the DCR and ORR.

### 2.4 Statistical Analysis

All statistical analyses were performed using IBM SPSS Statistics version 22.0 (IBM Corp, Armonk, NY, USA). Continuous variables were expressed as means with standard deviations, and comparisons between groups were conducted using two‐sample *t*‐tests. Categorical variables were presented as percentages, and the chi‐squared test was employed to compare differences between groups. All statistical analyses were two‐sided, with a significance level of 0.05.

The sample size calculation was based on the primary endpoint, DCR. Assuming a DCR of 80% in the control group and an anticipated improvement to 95% in the LMF group, with a two‐sided alpha level of 0.05 and 80% power, a total of 82 patients (41 per group) were required. This calculation accounted for an expected dropout rate of 10%.

## 3 Results

### 3.1 Baseline Characteristics of Patients

**FIGURE 1:**
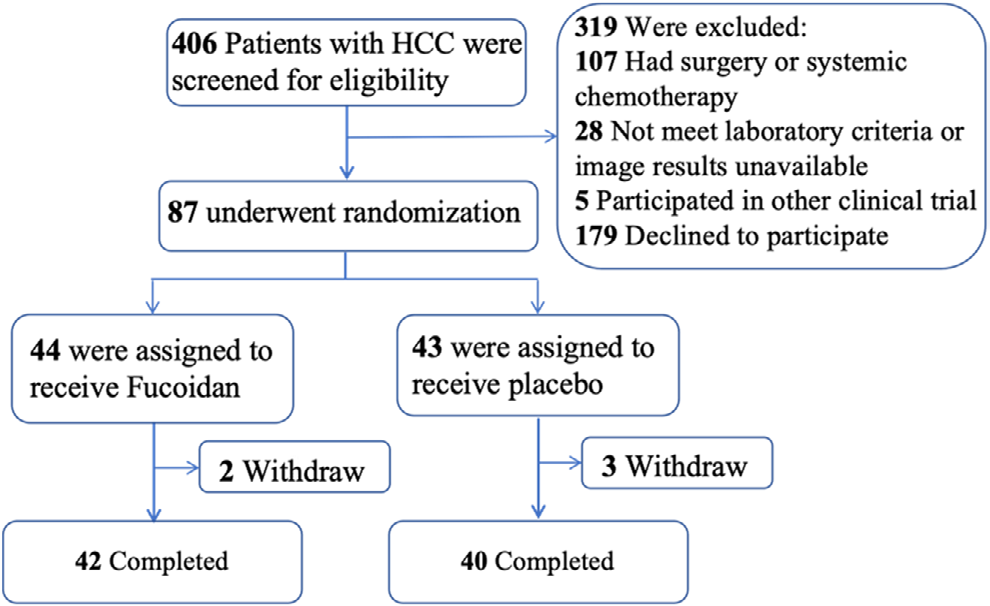
Patient enrolment, randomisation and follow‐up.

Between September 2019 and December 2022, 87 patients diagnosed with unresectable HCC were enrolled and randomised (Figure 1). Of these, one patient discontinued the 6‐month intervention prematurely, two were lost to follow‐up, one died from oesophageal and gastric variceal bleeding, and one died from severe pneumonia. These five patients were excluded from the per‐protocol analysis due to causes unrelated to HCC progression, TACE, or the investigational drug. Consequently, 82 patients were included in the final analysis, with 42 assigned to the LMF group and 40 to the placebo group.

Baseline demographic and clinical characteristics were well balanced between groups, including age, sex and pretreatment laboratory parameters such as white blood cell count, haemoglobin, platelet count, alpha‐fetoprotein (AFP), total bilirubin, albumin, prothrombin time, alanine aminotransferase (ALT), aspartate aminotransferase (AST) and gamma–glutamyl transferase (GGT) (Table 1). All patients were classified as BCLC Stage B, and none had macrovascular invasion or extrahepatic metastases. In addition, baseline ALBI scores were comparable between groups (LMF: −3.41 ± 0.45 vs. placebo: −3.53 ± 0.58; *p* = 0.31). No statistically significant differences were observed in any baseline characteristics, confirming appropriate group comparability for efficacy and safety analyses.

**TABLE 1:** Baseline demographic and clinical characteristics of patients in the LMF and placebo groups.

Characteristic	LMF group (*n* = 42)	Placebo group (*n* = 40)	*p*
Age (years), Mean ± SD	56.74 ± 11.14	60.63 ± 10.69	0.0833
Sex, *n* (%)			0.8228
Male	34 (80.95)	32 (80.0)	
Female	8 (19.05)	8 (20.0)	
Haematological parameters			
White blood cell count (×10^9^/L)	4.92 ± 1.70	4.93 ± 1.77	0.7935
Haemoglobin (g/L)	136.5 ± 17.0	133.1 ± 19.3	0.5204
Platelet count (×10^9^/L)	142.9 ± 73.0	138.0 ± 86.3	0.8360
Liver function and biomarkers			
Alpha‐fetoprotein (AFP) (ng/mL)	6815.66 ± 17892.58	6701.04 ± 15733.65	0.9591
Total bilirubin (μmol/L)	18.25 ± 11.49	15.24 ± 7.23	0.0686
Albumin (g/L)	40.1 ± 4.4	38.3 ± 4.6	0.5283
Prothrombin time (s)	12.76 ± 1.38	12.85 ± 1.29	0.7202
Liver enzymes			
Alanine aminotransferase (ALT) (U/L)	44.4 ± 25.1	35.6 ± 26.6	0.1544
Aspartate aminotransferase (AST) (U/L)	51.3 ± 27.7	58.9 ± 60.6	0.4551
Gamma–glutamyl transferase (GGT) (U/L)	160.7 ± 115.0	141.1 ± 122.7	0.5378
ALBI scores	−3.41 ± 0.45	−3.53 ± 0.58	0.3067

Abbreviations: AFP, alpha‐fetoprotein; ALB, albumin; ALT, alanine aminotransferase; AST, aspartate aminotransferase; GGT, gamma–glutamyl transferase; Hb, haemoglobin; PLT, platelet count; PT, prothrombin time; TBil, total bilirubin; WBC, white blood cell count.

### 3.2 Primary Outcome

The DCR, defined as the sum of CR, PR and SD rates, was significantly higher in the LMF group than in the placebo group (95.24% vs. 80.00%, respectively; *p* = 0.035, Table 2). The reduction in progressive disease (PD) in the LMF group (4.76%) compared with the placebo group (20.00%) suggests that LMF may contribute to delaying tumour progression.

**TABLE 2:** Disease control and objective response rates in the LMF and placebo groups.

Response category	Placebo group (*n* = 40), *n* (%)	LMF group (*n* = 42), *n* (%)	*χ* ^2^	*p*
Complete response (CR)	7 (17.5)	9 (21.4)	0.162	0.6873
Partial response (PR)	7 (17.5)	13 (31.0)	1.520	0.2176
Stable disease (SD)	18 (45.0)	18 (42.9)	0.021	0.8836
Progressive disease (PD)	8 (20.0)	2 (4.8)	3.901	0.0483
Objective response rate (ORR) (CR + PR)	14 (35.0)	22 (52.4)	2.513	0.1129
Disease control rate (DCR) (CR + PR + SD)	32 (80.0)	40 (95.2)	4.443	0.0350

Abbreviations: CR, complete response; DCR, disease control rate; ORR, objective response rate; PD, progressive disease; PR, partial response; SD, stable disease.

### 3.3 Secondary Outcome

Patients in the LMF group exhibited a trend towards a higher objective response rate (ORR), defined as the sum of CR and PR rates, compared with the placebo group (52.38% vs. 35.00%, respectively; *p* = 0.1129, Table 2), although this difference did not reach statistical significance.

Throughout the trial period, no severe adverse events were reported in either group. The rate of adverse events, including fever, headache, nausea, vomiting and leukopenia, was comparable between the two groups, with no significant treatment‐related toxicities observed. Additionally, no cases of treatment discontinuation or death due to treatment‐related toxicity were documented.

Quality‐of‐life assessments revealed no significant differences between the study and placebo groups in terms of limitations in daily activities, loss of appetite, constipation, sleep disturbances, anxiety or fatigue (Table 3). These findings confirm that the addition of LMF did not introduce any additional toxicity while maintaining a favourable safety profile. Despite the improved disease control rate in the LMF group, no measurable impact on patient‐reported quality‐of‐life parameters was observed.

**TABLE 3:** Quality‐of‐life outcomes in the LMF and placebo groups.

Quality‐of‐life domain	Placebo group (*n* = 40), *n* (%)	LMF group (*n* = 42), *n* (%)	*p*
Limited ability to perform daily activities			0.7504
Grade 1	31 (77.5)	35 (83.3)	
Grade 2	8 (20.0)	5 (11.9)	
Grade 3	1 (2.5)	2 (4.8)	
Grade 4	0 (0.0)	0 (0.0)	
Loss of appetite			0.2452
Grade 1	31 (77.5)	38 (90.5)	
Grade 2	9 (22.5)	3 (7.1)	
Grade 3	0 (0.0)	1 (2.4)	
Grade 4	0 (0.0)	0 (0.0)	
Constipation			0.9283
Grade 1	34 (85.0)	36 (85.7)	
Grade 2	6 (15.0)	6 (14.3)	
Grade 3	0 (0.0)	0 (0.0)	
Grade 4	0 (0.0)	0 (0.0)	
Sleep disturbances			0.5396
Grade 1	17 (42.5)	20 (47.6)	
Grade 2	21 (52.5)	21 (50.0)	
Grade 3	2 (5.0)	1 (2.4)	
Grade 4	0 (0.0)	0 (0.0)	
Anxiety			0.2436
Grade 1	29 (72.5)	35 (83.3)	
Grade 2	11 (27.5)	7 (16.7)	
Grade 3	0 (0.0)	0 (0.0)	
Grade 4	0 (0.0)	0 (0.0)	
Fatigue			0.1613
Grade 1	18 (45.0)	24 (57.1)	
Grade 2	20 (50.0)	18 (42.9)	
Grade 3	2 (5.0)	0 (0.0)	
Grade 4	0 (0.0)	0 (0.0)	

*Note:* Symptom severity is categorised according to a four‐grade scale: Grade 1: Mild symptoms, no intervention needed. Grade 2: Moderate symptoms, minimal intervention. Grade 3: Severe symptoms, significant interference. Grade 4: Life‐threatening or disabling symptoms.

### 3.4 Child‐Pugh Liver Function Class

At baseline, all patients were classified as Child–Pugh Class A, meeting the eligibility criteria for study enrolment. Posttreatment liver function was assessed using Child–Pugh classification at a fixed time point—6 months after initiation of LMF or placebo administration and at least 4 weeks after the last TACE procedure—to ensure recovery from acute treatment‐related hepatic stress.

Following treatment, a significantly greater proportion of patients in the LMF group‐maintained Child–Pugh Class A status compared with the placebo group (80.95% vs. 62.50%; *p* = 0.029, Table 4). Conversely, the proportion of patients who progressed to Child–Pugh Class B was higher in the placebo group (35.00%) than in the LMF group (14.29%), while Class C events remained rare in both arms. These results indicate that LMF may exert a hepatoprotective effect, potentially mitigating cumulative hepatic injury induced by repeated TACE procedures. Preservation of liver function is particularly important in patients undergoing locoregional therapy for unresectable HCC, as hepatic decompensation is a major determinant of treatment discontinuation and poor prognosis.

**TABLE 4:** Changes in Child–Pugh liver function classification before and after treatment in the LMF and placebo groups.

Child–Pugh classification	Placebo group (*n* = 40), *n* (%)	LMF group (*n* = 42), *n* (%)	*p*
Class A (Well‐compensated disease)	25 (62.5)	34 (80.9)	0.063
Class B (Significant functional compromise)	14 (35.0)	6 (14.3)	0.029
Class C (Decompensated liver disease)	1 (2.5)	2 (4.8)	0.586

*Note:* Liver function was assessed using Child–Pugh classification at baseline and at a fixed posttreatment time point—6 months after initiation of study intervention and at least four weeks following the final TACE procedure. A significantly higher proportion of patients in the LMF group‐maintained Child–Pugh class A status compared with the placebo group, suggesting a potential hepatoprotective effect of LMF during TACE‐based treatment. Preservation of liver function is a key determinant of continued treatment eligibility and long‐term outcomes in patients with unresectable HCC.

### 3.5 Intention‐To‐Treat (ITT) Analysis

To address potential concerns regarding exclusion of randomised patients, we conducted a supplementary intention‐to‐treat (ITT) analysis including all 87 patients originally randomised. In this analysis, five patients who did not complete the intervention were retained in their originally assigned groups: One patient in the LMF group who discontinued treatment prematurely, two patients (one in each group) who were lost to follow‐up and two patients (one per group) who died from causes unrelated to HCC, TACE or study medication (i.e., variceal bleeding and severe pneumonia).

Using the last observation carried forward (LOCF) approach, disease control status at the last available imaging assessment was carried forward for these patients. The ITT analysis yielded a disease control rate (DCR) of 93.02% in the LMF group and 78.05% in the placebo group (*p* = 0.041), consistent with the findings of the per‐protocol analysis. The objective response rate (ORR) also remained higher in the LMF group (51.16%) than in the placebo group (34.15%), though not statistically significant (*p* = 0.117).

These results confirm the robustness of the primary outcome and support the efficacy of LMF even when analysed under the more conservative ITT principle.

## 4 Discussion

This randomised, double‐blind, placebo‐controlled trial evaluated the efficacy and safety of LMF as an adjunct to TACE in patients with HCC that was not amenable to surgical resection or local ablative therapies. TACE, a locoregional therapy, is typically indicated for patients with HCC who are unsuitable for resection or local ablation [12, 13, 30]. Given that all enrolled patients had disease that precluded curative‐intent local treatment options, this study sought to determine whether LMF could enhance tumour control and improve clinical outcomes in this challenging patient population. The results demonstrated a significantly higher DCR in the LMF group compared with the placebo group (95.24% vs. 80.00%; *p* = 0.035), suggesting that LMF may enhance tumour control in this setting (Table 2). Although the ORR was higher in the LMF group (52.38% vs. 35.00%), the difference did not reach statistical significance (*p* = 0.1129). Importantly, the reduction in PD in the LMF group compared with the placebo group (4.76% vs. 20.00%) suggests that LMF may contribute to delaying tumour progression, which is clinically relevant given the high recurrence rates following TACE. These findings indicate a potential benefit of LMF in improving tumour response, warranting further investigation into its long‐term clinical efficacy and underlying mechanisms. To our knowledge, this is the first randomised, double‐blind, placebo‐controlled trial to evaluate LMF as an adjunct to TACE in unresectable HCC. It was specifically designed to address two major clinical challenges—achieving effective tumour control and mitigating TACE‐induced hepatotoxicity. The trial met its predefined primary endpoint, with a statistically significant improvement in DCR and preservation of Child–Pugh Class A liver function, thereby sustaining eligibility for repeated TACE. Importantly, these benefits were achieved without increasing adverse event incidence, supporting the favourable safety profile of LMF. Mechanistically, the findings align with LMF's proposed dual action—antiangiogenic and antitumour effects alongside hepatoprotection potentially mediated via modulation of the TGF‐β1/Smad pathway—providing biological plausibility and translational relevance for clinical integration [1, 17, 19, 20, 31, 32, 33, 34, 35, 36].

Baseline characteristics were well balanced between groups, minimising potential confounding factors (Table 1). Notably, no severe adverse events were reported, and no patients discontinued treatment due to toxicity (Table 3), supporting the safety and tolerability of LMF as an adjunctive therapy. The overall incidence of treatment‐related adverse events, including fever, headache, nausea, vomiting and leukopenia, was comparable between the two groups, confirming that the addition of LMF did not introduce additional toxicity. Child–Pugh liver function classification significantly improved in the LMF group following treatment (*p* = 0.029), raising the possibility that LMF may exert hepatoprotective effects (Table 4). A greater proportion of patients in the LMF group‐maintained Child–Pugh Class A status (80.95% vs. 62.50%), whereas more patients in the placebo group progressed to Child–Pugh Class B (35.00% vs. 14.29%). This preservation of liver function suggests that LMF may mitigate the hepatic toxicity commonly associated with repeated TACE procedures, which is a key limiting factor in its prolonged use. LMF mitigates hepatic toxicity through multiple mechanisms, primarily involving antioxidative, anti‐inflammatory, immune‐regulatory and signal pathway modulation effects [17, 35, 37]. It reduces oxidative stress by scavenging reactive oxygen species and enhancing antioxidant enzyme activity [17, 35, 37]. LMF suppresses inflammatory cytokines (e.g., IL‐1β, TNF‐α) by inhibiting NF‐κB activation [16, 33, 35, 36]. Additionally, it regulates immune responses by modulating macrophage polarisation and NK cell activity [16]. Moreover, LMF influences key signalling pathways such as TGF‐β1/Smad, SIRT1/AMPK/PGC1α and ASGR/STAT3/HNF4A, contributing to liver protection and fibrosis prevention [17, 33, 36]. These findings suggest that LMF could play a role in improving hepatic function in patients undergoing TACE, though further studies are needed to elucidate the biological mechanisms underlying these observations. Given that hepatic toxicity is a major limiting factor in the repeated administration of TACE, the ability of LMF to preserve liver function may enable more sustained locoregional therapy and ultimately improve overall survival (OS). Although this study was not designed to assess OS as a primary endpoint, it was prospectively planned with systematic survival status tracking for all participants, in accordance with a predefined follow‐up protocol. This ensured that long‐term endpoints such as OS and PFS could be captured as exploratory outcomes without deviating from the original trial design. As of the last follow‐up on June 30, 2025, the median OS was 26 months (95% CI: 18–32) in the LMF group and 25 months (95% CI: 17–31) in the placebo group. These exploratory findings, while limited by sample size, were derived from structured longitudinal follow‐up and provide an early signal suggestive of potential long‐term benefit. The stepwise approach—beginning with early efficacy assessment using DCR, which reached statistical significance in this trial, and progressing to formal evaluation of OS and PFS in adequately powered multicentre studies—is consistent with established trial methodology for developing and validating novel adjunctive strategies in intermediate‐stage HCC [23, 24, 25, 26, 27, 28, 38]. This framework allows early identification of promising therapeutic signals, while ensuring that subsequent trials are appropriately designed to confirm survival benefit and define the full clinical utility of LMF.

LMF, a sulfated polysaccharide derived from brown seaweed, exerts multiple antitumour effects that contribute to its potential as an adjunctive therapy for HCC treated with TACE [1, 16, 17, 18, 19]. The findings from this study suggest that LMF enhances DCR while mitigating hepatic toxicity, likely through its antiangiogenic, immune‐modulatory and antifibrotic properties [17, 35, 37]. The ability of LMF to inhibit HCC progression is supported by its role in suppressing tumour proliferation and inducing apoptosis [1, 19, 20]. Previous studies have demonstrated that LMF inhibits key oncogenic pathways, including the STAT3 signalling cascade, which is involved in cell survival and proliferation [1, 19, 20]. Additionally, LMF exerts anti‐angiogenic effects by downregulating hypoxia‐inducible factor‐1α (HIF‐1α) and vascular endothelial growth factor (VEGF), thereby restricting neovascularisation and nutrient supply to the tumour microenvironment [20, 31, 32, 33, 34, 35, 36]. By targeting both tumour growth and angiogenesis [1, 19, 20, 31, 32, 33, 34, 35, 36], LMF may complement the embolic effects of TACE, leading to improved tumour control. Beyond its direct effects on tumour biology, LMF enhances host immune responses, which may further contribute to its therapeutic efficacy [16, 21, 32, 39, 40]. Preclinical data suggest that LMF increases the activity of NK cells and macrophages [16, 21], promoting immune surveillance and cytotoxicity against tumour cells. This immunomodulatory effect is particularly relevant in the context of TACE [16, 21, 32, 39, 40], as ischaemic injury following embolization can induce an immunosuppressive microenvironment that facilitates tumour progression. By modulating immune function, LMF may help counteract these deleterious effects, thereby prolonging tumour control and improving clinical outcomes [16, 21, 32, 39, 40]. A key finding of our study is the potential hepatoprotective effect of LMF, as evidenced by improved Child–Pugh liver function classification in the LMF group. TACE is known to cause hepatic toxicity due to ischaemic insult and chemotherapy‐induced damage, which can compromise liver function and limit repeated treatment cycles [41]. LMF appears to mitigate these effects through its anti‐inflammatory and antifibrotic properties [17, 35, 37]. Specifically, LMF has been shown to inhibit the TGF‐β1/Smad signalling pathway, which is a central mediator of hepatic fibrosis [17]. By preventing excessive collagen deposition and fibrosis progression, LMF may help preserve liver function [17, 33], allowing patients to tolerate ongoing locoregional therapy and potentially improving long‐term survival. However, we acknowledge that these proposed hepatoprotective mechanisms are derived primarily from preclinical and prior clinical literature [16, 17, 20, 21, 31, 32, 33, 34, 35, 36, 37, 39, 40], and that our trial did not include correlative biomarker analyses to directly validate these pathways in patients. Future studies should incorporate prospective collection of serum fibrosis markers (e.g., hyaluronic acid, procollagen Type III N‐terminal peptide), imaging‐based fibrosis staging and, where feasible, liver biopsy samples for molecular pathway analysis to establish a causal link between LMF's biochemical effects and its clinical benefits in HCC patients undergoing TACE [42, 43]. Although overall survival (OS) was not the endpoint of this study, the observed trends suggest that prolonged follow‐up may reveal a survival benefit with LMF supplementation. The significant improvement in DCR (Table 2), combined with the preservation of hepatic function (Table 4), provides a strong rationale for further investigations into LMF's role in HCC management. Future studies should evaluate the long‐term effects of LMF in larger, multicentre cohorts and explore its potential integration with systemic therapies, such as immune checkpoint inhibitors, to optimise treatment outcomes in patients with unresectable HCC. In interpreting secondary endpoints, it is noteworthy that LMF improved objective hepatic reserve (ALBI and Child–Pugh) whereas patient‐reported QoL gains were less synchronous (Table 3). This pattern is consistent with existing literature showing significant correlations between QoL and objective liver function indices such as ALBI grade, and with ALBI being recognised as a sensitive and reproducible metric of hepatic reserve with prognostic relevance for post‐TACE outcomes [44, 45]. This discrepancy likely reflects differences in construct and timing: ALBI is a sensitive biochemical index of liver reserve, while QoL captures multidimensional symptoms that may be transiently affected by postembolisation syndrome and nonhepatic factors, with perceptible improvements emerging later. Moreover, QoL was an exploratory endpoint and underpowered in this study. Future trials should prespecify minimal clinically important differences (MCID) for QoL domains and include later assessments to better capture these changes. From a health‐economics perspective, compared with high‐cost tyrosine kinase inhibitor or immunotherapy combinations, LMF is an orally administered, well‐tolerated adjunct with lower financial burden and broader accessibility, supporting treatment continuity across varied healthcare settings [16, 21, 32, 39, 40]. These characteristics warrant future cost‐effectiveness analyses and evaluation of LMF in combination with TACE and systemic agents to maintain hepatic reserve and treatment eligibility.

The results of this trial challenge the conventional paradigm that TACE must be used in isolation or solely in combination with systemic therapies such as tyrosine kinase inhibitors [12, 14, 15]. By demonstrating that a natural compound with antiangiogenic, immunomodulatory and antifibrotic properties can enhance the efficacy of TACE while mitigating hepatic toxicity, this study introduces a novel treatment strategy that warrants further investigation. Given the safety profile of LMF and its potential role in both tumour control and hepatic protection, further research should focus on identifying optimal patient populations, refining dosing strategies and integrating LMF with evolving systemic therapies to maximise its clinical impact. Future research should focus on longer‐term survival studies, mechanistic evaluations of LMF's hepatoprotective effects and the potential for combination regimens incorporating systemic immunotherapies.

This randomised, double‐blind, placebo‐controlled trial provides robust evidence supporting the potential role of LMF as an adjunct to TACE in patients with unresectable HCC. One of the major strengths of this study is its rigorous design, including strict eligibility criteria, comprehensive baseline assessments and standardised TACE protocols, ensuring homogeneity in the study population. The randomisation process effectively minimised selection bias, while the double‐blind approach reduced the risk of observer and reporting bias. Furthermore, the inclusion of a well‐defined primary endpoint—the DCR—allowed for a clinically meaningful assessment of treatment efficacy, while secondary endpoints, including ORR and Child–Pugh liver function classification, provided additional insights into tumour response and hepatic preservation. Importantly, the study demonstrated a statistically significant improvement in DCR with LMF supplementation, suggesting its potential to enhance tumour control. Additionally, the observation that LMF was associated with improved hepatic function supports its hepatoprotective effects, which may allow for prolonged administration of TACE without compromising liver reserve.

Despite these strengths, several limitations should be acknowledged. First, the relatively small sample size may have limited statistical power for certain outcomes, particularly OS, where a nonsignificant trend favouring LMF was observed. The trial was prospectively designed with DCR as the primary endpoint, a sensitive and clinically relevant measure for early efficacy, hepatic function preservation and TACE eligibility maintenance [23, 24, 25, 26, 27, 28, 38]. As prespecified, this endpoint was met with statistical significance (95.24% vs. 80.00%, *p* = 0.035). Survival status was systematically tracked from study entry, allowing exploratory analyses: as of June 30, 2025, median OS was 26 months (95% CI, 18–32) for LMF and 25 months (95% CI, 17–31) for placebo. While underpowered for OS/PFS, these findings provide an early signal supporting further evaluation in larger, survival‐powered trials. Second, although no significant difference in QoL was detected, the study was not powered for this secondary endpoint; QoL was nevertheless assessed systematically at prespecified intervals, and these exploratory data will inform effect size assumptions for future adequately powered trials. Third, while LMF significantly improved Child‐Pugh classification, mechanistic validation is needed; although preclinical studies support pathways such as TGF‐β1/Smad, STAT3 and SIRT1/AMPK/PGC‐1α, this trial did not include full biomarker profiling. Future studies should integrate feasible correlative endpoints, such as serum fibrosis markers and imaging‐based fibrosis staging, to substantiate these effects in patients. Finally, this single‐centre design, enrolling exclusively BCLC‐B, TACE‐naïve patients, was intentional to ensure baseline homogeneity and minimise confounding from prior TACE. This approach enhances internal validity and procedural consistency but limits generalisability. Future multicentre trials across diverse practice settings will be essential to confirm these findings and determine whether benefits persist in more heterogeneous populations. To establish the long‐term clinical value of LMF and to elucidate the biological pathways underlying its effects, future investigations should be designed with sufficient statistical power to detect differences in overall and progression‐free survival, while incorporating prespecified minimal clinically important differences for quality‐of‐life domains, longitudinal assessment of ALBI and Child–Pugh trajectories, and health‐economic outcomes. Mechanistic insight will require prospective integration of biomarker analyses, including serum markers of fibrosis and hepatic injury (such as TGF‐β1, PIIINP and hyaluronic acid), signalling and metabolic pathways (p‐STAT3, SIRT1/AMPK/PGC‐1α), immune profiling and noninvasive imaging‐based fibrosis staging, with mediation analyses to clarify causal links between biological changes and clinical benefit. In parallel, randomised trials combining LMF with tyrosine kinase inhibitors or PD‐1/PD‐L1 blockade in conjunction with TACE should evaluate not only survival and hepatic function preservation but also patient‐reported outcomes, safety and cost‐effectiveness. Collectively, these strategies will enable rigorous multicentre validation and define the role of LMF within an integrated therapeutic framework for intermediate‐stage HCC. Future research should focus on three priorities: conducting survival‐powered, multicentre trials with prespecified quality‐of‐life and health‐economic endpoints; embedding mechanistic studies with prospective biomarker and imaging‐based fibrosis profiling to link biological effects to clinical outcomes; and testing LMF in rational combinations with tyrosine kinase inhibitors or PD‐1/PD‐L1 blockade atop TACE. These steps will clarify efficacy, mechanism and optimal integration of LMF into HCC management.

## 5 Conclusion

This randomised, double‐blind, placebo‐controlled trial provides the first clinical evidence that LMF enhances TACE efficacy in unresectable HCC. LMF significantly improved the disease control rate and preserved hepatic function, mitigating TACE‐related toxicity. Although not powered to assess overall survival, the observed trend suggests potential long‐term benefits. With a favourable safety profile, LMF represents a promising adjunct to TACE. Further large‐scale, multicentre trials are needed to confirm its role in optimising HCC treatment.

## Author Contributions

Conceptualization and Study Design: Yanting Zou, Szu‐Yuan Wu, Xudong Qu, Qunyan Yao. Data Collection and Clinical Investigations: Wanqin Zhang, Xudong Qu, Wei Zhang, Xizhong Shen, Qunyan Yao. Data Analysis and Interpretation: Yanting Zou, Szu‐Yuan Wu, Wanqin Zhang, Xudong Qu. Manuscript Drafting and Critical Revision: Yanting Zou, Szu‐Yuan Wu, Xudong Qu, Qunyan Yao. Supervision and Project Administration: Xudong Qu, Qunyan Yao, Szu‐Yuan Wu. All authors have reviewed and approved the final version of the manuscript and agree to be accountable for all aspects of the work.

## Ethics Statement

This study was conducted in accordance with the Declaration of Helsinki and Good Clinical Practice (GCP) guidelines. The trial was approved by the Institutional Review Board (IRB) of Zhongshan Hospital, Fudan University (Approval Number: [IRB Number: B2018‐252(4)R]) and was registered at ClinicalTrials.gov (NCT04066660).

## Consent

Written informed consent was obtained from all participants before enrolment.

## Conflicts of Interest

The authors declare no conflicts of interest.

## Supporting information


**Data S1:** liv70347‐sup‐0001‐Supinfo.docx.

## Data Availability

The data sets used and analysed during the current study are available from the corresponding author upon reasonable request. Due to ethical and regulatory restrictions, individual patient data will not be made publicly available to ensure participant confidentiality.
