# Supplementary material for: Fucoidan Improves Tumour Control and Liver Function in TACE for Unresectable Hepatocellular Carcinoma: A Randomised Trial
Source: Liver Int. 2025 Sep 17;45(10):e70347. doi: 10.1111/liv.70347 (PMC12442522; doi:10.1111/liv.70347)
Supplement: Supplementary file 1 — Data S1: liv70347‐sup‐0001‐Supinfo.docx. [file LIV-45-0-s001.docx]

# CONSORT 2010 Checklist of Information to Include

| Section/Topic | Item No | Checklist item | Reported on page No |
| --- | --- | --- | --- |
| Title and abstract | 1a | Identification as a randomized trial in the title | Yes |
| Title and abstract | 1b | Structured summary of trial design, methods, results, and conclusions | Yes |
| Introduction | 2a | Scientific background and explanation of rationale | Yes |
| Introduction | 2b | Specific objectives or hypotheses | Yes |
| Methods | 3a | Description of trial design (such as parallel, factorial) | Yes |
| Methods | 4a | Eligibility criteria for participants | Yes |
| Methods | 5 | The interventions for each group | Yes |
| Methods | 6a | Completely defined primary and secondary outcome measures | Yes |
| Methods | 7a | How sample size was determined | Yes |
| Methods | 8a | Method used to generate the random allocation sequence | Yes |
| Methods | 9 | Allocation concealment mechanism | Yes |
| Methods | 10 | Who generated the allocation sequence, enrolled participants, and assigned interventions | Yes |
| Results | 13a | Participant flow (diagram is strongly recommended) | Yes |
| Results | 14a | Dates defining periods of recruitment and follow-up | Yes |
| Results | 15 | Baseline demographic and clinical characteristics | Yes |
| Results | 17a | Outcomes and estimation | Yes |
| Discussion | 20 | Interpretation consistent with results, balancing benefits and harms | Yes |
| Other | 23 | Registration number and name of trial registry | Yes |
| Other | 25 | Sources of funding and other support | Yes |
